# *catena*-Poly[[aqua­[1,4-bis­(1*H*-imidazol-4-yl)benzene]cadmium]-μ_3_-5-methyl­isophthalato]

**DOI:** 10.1107/S1600536811030509

**Published:** 2011-08-02

**Authors:** Sen-Lin Yang, De-Hai Wang, Shui-Sheng Chen

**Affiliations:** a Department of Chemistry, Fuyang Normal College, Fuyang, Anhui 236041, People’s Republic of China

## Abstract

In the title coordination polymer, [Cd(C_9_H_6_O_4_)(C_12_H_10_N_4_)(H_2_O)]_*n*_, the Cd^II^ atom has a NO_6_ donor set and is coord­inated by five carboxyl­ate O atoms from three different 5-methyl-1,3-phenyl­enediacetate (pda^2−^) anions, one O atom from a water mol­ecule and one N atom from a 1,4-bis­(1*H*-imidazol-4-yl)benzene (*L*) ligand, displaying a highly distorted penta­gonal–bipyramidal geometry. Each pda^2−^ anion acts as a μ_3_-bridge, linking Cd^II^ atoms to form one-dimensional slabs extending parallel to [010]. In the crystal, adjacent mol­ecules are linked through N—H⋯N and N—H⋯O hydrogen bonds into a three-dimensional network.

## Related literature

For background to metal-organic hybrid materials, see: Bradshaw *et al.* (2005 ▶); Ockwig *et al.* (2005 ▶). For structures containing mixed ligands, see: Liu *et al.* (2007 ▶); Chen *et al.* (2006 ▶); Choi & Jeon (2003 ▶). For related structures, see: Chen *et al.* (2010 ▶; 2011 ▶).
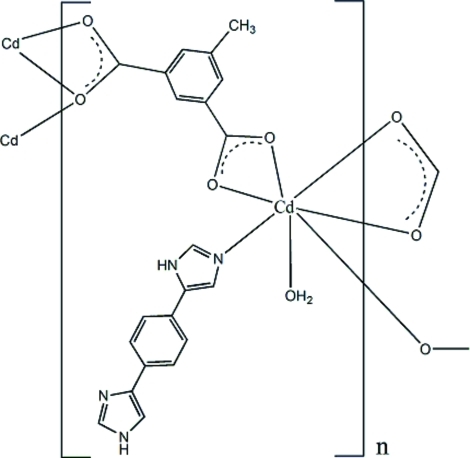


## Experimental

### 

#### Crystal data

[Cd(C_9_H_6_O_4_)(C_12_H_10_N_4_)(H_2_O)]
*M* *_r_* = 518.80
Triclinic, 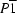

*a* = 6.9407 (9) Å
*b* = 9.8231 (13) Å
*c* = 15.506 (2) Å
α = 74.091 (2)°
β = 85.963 (2)°
γ = 70.707 (2)°
*V* = 959.4 (2) Å^3^
*Z* = 2
Mo *K*α radiation
μ = 1.18 mm^−1^
*T* = 296 K
0.18 × 0.16 × 0.12 mm

#### Data collection

Bruker APEXII CCD diffractometer
Absorption correction: multi-scan (*SADABS*; Sheldrick, 1996 ▶) *T*_min_ = 0.815, *T*_max_ = 0.871
15863 measured reflections
4376 independent reflections
4161 reflections with *I* > 2σ(*I*)
*R*_int_ = 0.022

#### Refinement

*R*[*F* ^2^ > 2σ(*F* ^2^)] = 0.026
*wR*(*F* ^2^) = 0.128
*S* = 1.09
4376 reflections
281 parameters
H-atom parameters constrained
Δρ_max_ = 0.86 e Å^−3^
Δρ_min_ = −0.80 e Å^−3^

### 

Data collection: *APEX2* (Bruker, 2008 ▶); cell refinement: *SAINT* (Bruker, 2008 ▶); data reduction: *SAINT*; program(s) used to solve structure: *SHELXS97* (Sheldrick, 2008 ▶); program(s) used to refine structure: *SHELXL97* (Sheldrick, 2008 ▶); molecular graphics: *XP* in *SHELXTL* (Sheldrick, 2008 ▶) and *DIAMOND* (Brandenburg, 2000 ▶); software used to prepare material for publication: *SHELXTL*.

## Supplementary Material

Crystal structure: contains datablock(s) global, I. DOI: 10.1107/S1600536811030509/wm2517sup1.cif

Structure factors: contains datablock(s) I. DOI: 10.1107/S1600536811030509/wm2517Isup2.hkl

Additional supplementary materials:  crystallographic information; 3D view; checkCIF report

## Figures and Tables

**Table 1:** Selected bond lengths (Å)

Cd1—N1	2.222 (3)
Cd1—O1^i^	2.315 (3)
Cd1—O3^ii^	2.380 (3)
Cd1—O4^ii^	2.404 (2)
Cd1—O2^i^	2.473 (3)
Cd1—O5	2.520 (3)
Cd1—O3	2.539 (3)

Symmetry codes: (i) 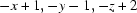; (ii) 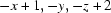.

**Table 2:** Hydrogen-bond geometry (Å, °)

*D*—H⋯*A*	*D*—H	H⋯*A*	*D*⋯*A*	*D*—H⋯*A*
N2—H2*A*⋯N4^iii^	0.86	2.17	2.975 (4)	157
N3—H3⋯O4^iv^	0.86	2.03	2.815 (4)	151

Symmetry codes: (iii) 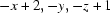; (iv) 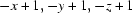.

**Fig. 1.**
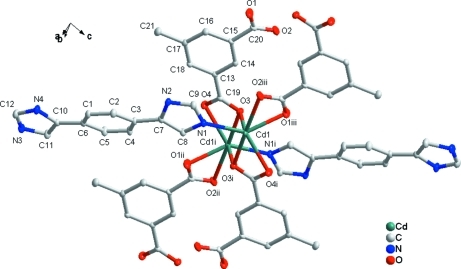
The coordination of the metal atom in compound (I). Displacement ellipsoids are drawn at the 30% probability level. [Symmetry codes: (i) 1 - x, -y, 2 - z (ii) x, 1 + y, z (iii) 1 - x, -1 - y, 2 - z.]

**Fig. 2.**
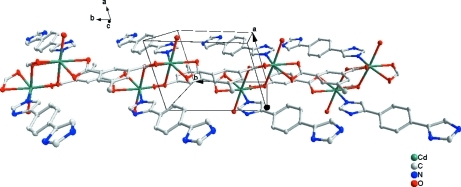
The slab formed from CdII atoms and pda2- anions. Displacement ellipsoids are drawn at 30% probability level.

**Fig. 3.**
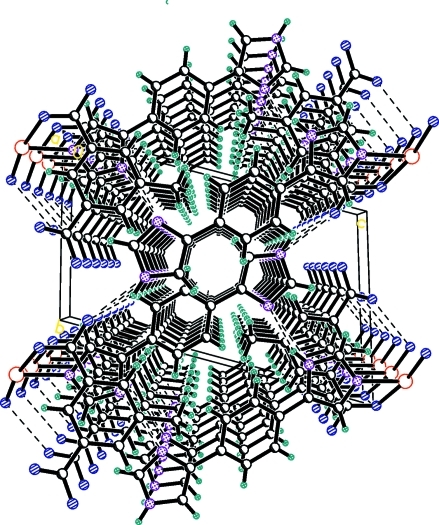
The three-dimensional network formed by hydrogen bonding interactions in the structure of compound (I).

## supplementary crystallographic information

### Comment

The rational design and synthesis of metal-organic frameworks (MOFs) has attracted considerable attention, which is stimulated by their intriguing aesthetic structures and topological features as well as their potential applications as materials (Bradshaw *et al.*, 2005; Ockwig *et al.*, 2005). The choice of suitable ligands is a key factor that greatly affects the structure and stabilization of the coordination architecture (Choi & Jeon 2003). For a more tunable ligand design mixed polycarboxylate and N-containing compounds (Liu *et al.*, 2007; Chen *et al.*, 2006) are favourable. Therefore we have focused on constructing complexes based on the organic ligand 1,4-di(1*H*-imidazol-4-yl)benzene (*L*) and polycarboxylate anions (Chen *et al.*, 2010; 2011). As an extension of our work, we report the synthesis and structure of a new Cd^II^ complex (I), which was obtained by solvothermal reaction of CdI_2_ with *L* and 5-methylisophthalic acid (H_2_pda).

The asymmetric unit of (I) consists of one Cd^II^ atom, one *L* ligaqnd, one pda^2-^ anion and one coordinated water molecule. Each Cd^II^ atom has a NO_6_ donor set and is coordinated by five carboxylate oxygen atoms from three different pda^2-^ anions, one water oxygen atom and one nitrogen atom from *L*, displaying a highly distorted pentagonal-bipyramidal geometry (Fig. 1). The pda^2-^ ligand acts as a µ_3_– bridge with two monodentate carboxylate groups to form one-dimensional slabs parellel to  [010] (Fig. 2). In the crystal, adjacent molecules are linked through N—H···N and N—H···O hydrogen bonding interactions into a three-dimensional network (Fig. 3).

### Experimental

All reagents and solvents were used as obtained commercially without further purification. A mixture containing CdI_2_ (36.6 mg, 0.1 mmol), *L* (21.0 mg, 0.1 mmol), H_2_pda (18.0 mg, 0.1 mmol), DMF (*N*:*N*'- dimethylformamide, 1 ml), 10 ml H_2_O was sealed in a 16 ml Teflon-lined stainless steel container and heated at 453 K for 72 h. After cooling to room temperature within 12 h, colorless crystals of (I) suitable for X-ray diffraction analysis were obtained in 48% Yield.

### Refinement

H atoms bonded to C atoms were placed geometrically and treated as riding, with C—H distances 0.93 Å and 0.96 Å for aryl and methyl type H-atoms, respectively with *U*_iso_(H) = 1.2*U*_eq_(C) or *U*_iso_(H) = 1.5*U*_eq_(C_methyl_). The amide H atoms were located from difference maps and refined with the N—H distances restrained to 0.86 Å and *U*_iso_(H) = 1.2*U*_eq_(N). The hydrogen atoms of the coordinated water molecule could not be located and thus were not included in the refinement.

### Crystal data

**Table:**

[Cd(C_9_H_6_O_4_)(C_12_H_10_N_4_)(H_2_O)]	*Z* = 2
*M**_r_* = 518.80	*F*(000) = 516
Triclinic, *P*1	*D*_x_ = 1.789 Mg m^−^^3^
Hall symbol: -P 1	Mo *K*α radiation, λ = 0.71073 Å
*a* = 6.9407 (9) Å	Cell parameters from 9986 reflections
*b* = 9.8231 (13) Å	θ = 2.7–27.6°
*c* = 15.506 (2) Å	µ = 1.18 mm^−^^1^
α = 74.091 (2)°	*T* = 296 K
β = 85.963 (2)°	Block, colorless
γ = 70.707 (2)°	0.18 × 0.16 × 0.12 mm
*V* = 959.4 (2) Å^3^	

### Data collection

**Table:**

Bruker APEXII CCD diffractometer	4376 independent reflections
Radiation source: fine-focus sealed tube	4161 reflections with *I* > 2σ(*I*)
graphite	*R*_int_ = 0.022
φ and ω scans	θ_max_ = 27.6°, θ_min_ = 1.4°
Absorption correction: multi-scan (*SADABS*; Sheldrick, 1996)	*h* = −5→9
*T*_min_ = 0.815, *T*_max_ = 0.871	*k* = −12→12
15863 measured reflections	*l* = −20→20

### Refinement

**Table:**

Refinement on *F*^2^	Primary atom site location: structure-invariant direct methods
Least-squares matrix: full	Secondary atom site location: difference Fourier map
*R*[*F*^2^ > 2σ(*F*^2^)] = 0.026	Hydrogen site location: inferred from neighbouring sites
*wR*(*F*^2^) = 0.128	H-atom parameters constrained
*S* = 1.09	*w* = 1/[σ^2^(*F*_o_^2^) + (0.098*P*)^2^ + 0.8397*P*] where *P* = (*F*_o_^2^ + 2*F*_c_^2^)/3
4376 reflections	(Δ/σ)_max_ = 0.002
281 parameters	Δρ_max_ = 0.86 e Å^−^^3^
0 restraints	Δρ_min_ = −0.80 e Å^−^^3^

### Special details

**Table:**

Geometry. All e.s.d.'s (except the e.s.d. in the dihedral angle between two l.s. planes) are estimated using the full covariance matrix. The cell e.s.d.'s are taken into account individually in the estimation of e.s.d.'s in distances, angles and torsion angles; correlations between e.s.d.'s in cell parameters are only used when they are defined by crystal symmetry. An approximate (isotropic) treatment of cell e.s.d.'s is used for estimating e.s.d.'s involving l.s. planes.
Refinement. Refinement of *F*^2^ against ALL reflections. The weighted *R*-factor *wR* and goodness of fit *S* are based on *F*^2^, conventional *R*-factors *R* are based on *F*, with *F* set to zero for negative *F*^2^. The threshold expression of *F*^2^ > σ(*F*^2^) is used only for calculating *R*-factors(gt) *etc*. and is not relevant to the choice of reflections for refinement. *R*-factors based on *F*^2^ are statistically about twice as large as those based on *F*, and *R*- factors based on ALL data will be even larger.

### Fractional atomic coordinates and isotropic or equivalent isotropic displacement parameters (Å^2^)

**Table:**

	*x*	*y*	*z*	*U*_iso_*/*U*_eq_	
Cd1	0.70159 (3)	−0.20380 (2)	1.035182 (12)	0.02480 (12)	
C1	0.9950 (5)	0.1669 (4)	0.5262 (2)	0.0261 (6)	
H1	1.0841	0.1400	0.4815	0.031*	
C2	0.9908 (5)	0.0593 (4)	0.6057 (2)	0.0267 (6)	
H2	1.0746	−0.0391	0.6127	0.032*	
C3	0.8630 (5)	0.0969 (3)	0.6747 (2)	0.0241 (6)	
C4	0.7404 (5)	0.2448 (4)	0.6627 (2)	0.0311 (7)	
H4	0.6565	0.2726	0.7087	0.037*	
C5	0.7415 (6)	0.3520 (4)	0.5827 (2)	0.0328 (7)	
H5	0.6569	0.4503	0.5756	0.039*	
C6	0.8676 (5)	0.3143 (3)	0.5130 (2)	0.0243 (6)	
C7	0.8556 (4)	−0.0187 (3)	0.7570 (2)	0.0226 (5)	
C8	0.7980 (5)	−0.0084 (3)	0.8415 (2)	0.0262 (6)	
H8	0.7546	0.0796	0.8597	0.031*	
C9	0.8833 (5)	−0.2419 (3)	0.8451 (2)	0.0254 (6)	
H9	0.9102	−0.3448	0.8648	0.031*	
C10	0.8657 (5)	0.4250 (3)	0.4273 (2)	0.0246 (6)	
C11	0.7830 (6)	0.5769 (4)	0.4057 (2)	0.0338 (7)	
H11	0.7115	0.6347	0.4433	0.041*	
C12	0.9308 (6)	0.5092 (4)	0.2888 (2)	0.0350 (7)	
H12	0.9784	0.5146	0.2308	0.042*	
C13	0.3642 (4)	−0.1324 (3)	0.83307 (19)	0.0197 (5)	
C14	0.3608 (4)	−0.2719 (3)	0.88386 (19)	0.0213 (5)	
H14	0.3291	−0.2864	0.9443	0.026*	
C15	0.4045 (4)	−0.3895 (3)	0.8449 (2)	0.0212 (5)	
C16	0.4601 (5)	−0.3674 (3)	0.7544 (2)	0.0248 (6)	
H16	0.4972	−0.4477	0.7290	0.030*	
C17	0.4607 (5)	−0.2279 (3)	0.7021 (2)	0.0236 (6)	
C18	0.4118 (4)	−0.1095 (3)	0.7421 (2)	0.0216 (5)	
H18	0.4110	−0.0152	0.7081	0.026*	
C19	0.3244 (4)	−0.0102 (3)	0.8788 (2)	0.0219 (5)	
C20	0.3822 (5)	−0.5364 (3)	0.8978 (2)	0.0263 (6)	
C21	0.5132 (6)	−0.2039 (4)	0.6041 (2)	0.0337 (7)	
H21A	0.5255	−0.1060	0.5810	0.050*	
H21B	0.4073	−0.2125	0.5714	0.050*	
H21C	0.6403	−0.2780	0.5977	0.050*	
N1	0.8142 (4)	−0.1499 (3)	0.89632 (18)	0.0255 (5)	
N2	0.9098 (4)	−0.1684 (3)	0.76096 (17)	0.0245 (5)	
H2A	0.9531	−0.2078	0.7172	0.029*	
N3	0.8263 (5)	0.6276 (3)	0.3174 (2)	0.0344 (6)	
H3	0.7919	0.7195	0.2862	0.041*	
N4	0.9599 (5)	0.3820 (3)	0.35234 (18)	0.0317 (6)	
O1	0.4754 (5)	−0.6519 (3)	0.87238 (19)	0.0408 (6)	
O2	0.2711 (4)	−0.5384 (3)	0.9641 (2)	0.0344 (6)	
O3	0.3531 (4)	−0.0476 (3)	0.96292 (17)	0.0282 (5)	
O4	0.2703 (4)	0.1241 (3)	0.83420 (17)	0.0345 (5)	
O5	1.0670 (4)	−0.3593 (3)	1.07976 (18)	0.0375 (6)	

### Atomic displacement parameters (Å^2^)

**Table:**

	*U*^11^	*U*^22^	*U*^33^	*U*^12^	*U*^13^	*U*^23^
Cd1	0.04272 (18)	0.01512 (16)	0.01673 (15)	−0.01121 (11)	0.00698 (10)	−0.00380 (10)
C1	0.0299 (14)	0.0252 (15)	0.0192 (13)	−0.0072 (12)	0.0048 (11)	−0.0026 (11)
C2	0.0300 (14)	0.0203 (14)	0.0218 (14)	−0.0019 (11)	0.0034 (11)	−0.0013 (11)
C3	0.0292 (14)	0.0218 (14)	0.0177 (13)	−0.0080 (11)	0.0024 (11)	−0.0004 (11)
C4	0.0399 (17)	0.0245 (15)	0.0242 (15)	−0.0078 (13)	0.0128 (13)	−0.0051 (12)
C5	0.0430 (17)	0.0196 (14)	0.0275 (16)	−0.0039 (13)	0.0088 (13)	−0.0028 (12)
C6	0.0289 (14)	0.0229 (14)	0.0190 (13)	−0.0095 (11)	−0.0005 (11)	−0.0007 (11)
C7	0.0247 (13)	0.0199 (13)	0.0205 (13)	−0.0078 (10)	0.0034 (10)	−0.0013 (11)
C8	0.0343 (15)	0.0197 (14)	0.0225 (14)	−0.0081 (11)	0.0046 (11)	−0.0042 (11)
C9	0.0303 (14)	0.0192 (13)	0.0212 (14)	−0.0040 (11)	0.0037 (11)	−0.0022 (11)
C10	0.0309 (14)	0.0219 (14)	0.0207 (14)	−0.0108 (11)	0.0008 (11)	−0.0023 (11)
C11	0.0473 (19)	0.0228 (15)	0.0242 (15)	−0.0069 (13)	0.0038 (13)	−0.0013 (12)
C12	0.053 (2)	0.0300 (17)	0.0193 (15)	−0.0135 (15)	0.0011 (14)	−0.0014 (13)
C13	0.0239 (12)	0.0148 (12)	0.0210 (13)	−0.0068 (10)	−0.0010 (10)	−0.0047 (10)
C14	0.0256 (13)	0.0186 (13)	0.0170 (12)	−0.0055 (10)	0.0010 (10)	−0.0027 (10)
C15	0.0270 (13)	0.0142 (12)	0.0230 (14)	−0.0098 (10)	−0.0012 (10)	−0.0017 (10)
C16	0.0302 (14)	0.0202 (14)	0.0239 (14)	−0.0060 (11)	0.0038 (11)	−0.0088 (11)
C17	0.0280 (13)	0.0233 (14)	0.0192 (13)	−0.0074 (11)	0.0011 (10)	−0.0063 (11)
C18	0.0271 (13)	0.0170 (12)	0.0205 (13)	−0.0090 (10)	0.0001 (10)	−0.0021 (10)
C19	0.0268 (13)	0.0170 (13)	0.0237 (14)	−0.0083 (10)	0.0015 (10)	−0.0071 (11)
C20	0.0380 (16)	0.0144 (13)	0.0265 (15)	−0.0121 (11)	−0.0059 (12)	0.0006 (11)
C21	0.0416 (17)	0.0353 (18)	0.0250 (16)	−0.0131 (14)	0.0069 (13)	−0.0104 (13)
N1	0.0338 (13)	0.0205 (12)	0.0194 (12)	−0.0088 (10)	0.0043 (10)	−0.0019 (10)
N2	0.0289 (12)	0.0222 (12)	0.0177 (11)	−0.0036 (10)	0.0036 (9)	−0.0043 (9)
N3	0.0479 (16)	0.0234 (13)	0.0248 (14)	−0.0109 (12)	−0.0001 (12)	0.0040 (11)
N4	0.0429 (15)	0.0265 (14)	0.0190 (12)	−0.0070 (11)	0.0041 (11)	−0.0014 (11)
O1	0.0698 (18)	0.0153 (11)	0.0350 (13)	−0.0136 (11)	0.0124 (13)	−0.0058 (10)
O2	0.0423 (13)	0.0209 (12)	0.0370 (14)	−0.0133 (10)	0.0063 (11)	−0.0004 (10)
O3	0.0406 (13)	0.0226 (11)	0.0226 (12)	−0.0102 (9)	0.0013 (9)	−0.0081 (9)
O4	0.0589 (15)	0.0151 (10)	0.0270 (12)	−0.0103 (10)	0.0033 (11)	−0.0042 (9)
O5	0.0406 (13)	0.0366 (14)	0.0313 (13)	−0.0107 (11)	0.0052 (10)	−0.0063 (11)

### Geometric parameters (Å, °)

**Table:**

Cd1—N1	2.222 (3)	C11—H11	0.9300
Cd1—O1^i^	2.315 (3)	C12—N3	1.325 (5)
Cd1—O3^ii^	2.380 (3)	C12—N4	1.326 (4)
Cd1—O4^ii^	2.404 (2)	C12—H12	0.9300
Cd1—O2^i^	2.473 (3)	C13—C14	1.388 (4)
Cd1—O5	2.520 (3)	C13—C18	1.399 (4)
Cd1—O3	2.539 (3)	C13—C19	1.497 (4)
Cd1—C20^i^	2.716 (3)	C14—C15	1.384 (4)
Cd1—C19^ii^	2.735 (3)	C14—H14	0.9300
C1—C6	1.392 (4)	C15—C16	1.407 (4)
C1—C2	1.392 (4)	C15—C20	1.503 (4)
C1—H1	0.9300	C16—C17	1.389 (4)
C2—C3	1.391 (4)	C16—H16	0.9300
C2—H2	0.9300	C17—C18	1.399 (4)
C3—C4	1.388 (4)	C17—C21	1.510 (4)
C3—C7	1.469 (4)	C18—H18	0.9300
C4—C5	1.391 (5)	C19—O4	1.252 (4)
C4—H4	0.9300	C19—O3	1.266 (4)
C5—C6	1.394 (5)	C19—Cd1^ii^	2.735 (3)
C5—H5	0.9300	C20—O2	1.241 (5)
C6—C10	1.465 (4)	C20—O1	1.260 (4)
C7—C8	1.364 (4)	C20—Cd1^i^	2.716 (3)
C7—N2	1.377 (4)	C21—H21A	0.9600
C8—N1	1.389 (4)	C21—H21B	0.9600
C8—H8	0.9300	C21—H21C	0.9600
C9—N1	1.315 (4)	N2—H2A	0.8600
C9—N2	1.340 (4)	N3—H3	0.8600
C9—H9	0.9300	O1—Cd1^i^	2.315 (3)
C10—C11	1.362 (5)	O2—Cd1^i^	2.473 (3)
C10—N4	1.392 (4)	O3—Cd1^ii^	2.380 (3)
C11—N3	1.371 (5)	O4—Cd1^ii^	2.404 (2)
			
N1—Cd1—O1^i^	142.24 (10)	N2—C9—H9	124.3
N1—Cd1—O3^ii^	88.65 (10)	C11—C10—N4	109.0 (3)
O1^i^—Cd1—O3^ii^	121.73 (9)	C11—C10—C6	129.7 (3)
N1—Cd1—O4^ii^	133.01 (10)	N4—C10—C6	121.3 (3)
O1^i^—Cd1—O4^ii^	84.74 (9)	C10—C11—N3	106.4 (3)
O3^ii^—Cd1—O4^ii^	54.72 (8)	C10—C11—H11	126.8
N1—Cd1—O2^i^	93.84 (10)	N3—C11—H11	126.8
O1^i^—Cd1—O2^i^	54.45 (9)	N3—C12—N4	112.3 (3)
O3^ii^—Cd1—O2^i^	175.37 (8)	N3—C12—H12	123.8
O4^ii^—Cd1—O2^i^	125.04 (9)	N4—C12—H12	123.8
N1—Cd1—O5	86.01 (9)	C14—C13—C18	120.3 (3)
O1^i^—Cd1—O5	101.86 (10)	C14—C13—C19	118.4 (3)
O3^ii^—Cd1—O5	109.61 (10)	C18—C13—C19	121.2 (3)
O4^ii^—Cd1—O5	81.20 (9)	C15—C14—C13	120.2 (3)
O2^i^—Cd1—O5	74.49 (10)	C15—C14—H14	119.9
N1—Cd1—O3	84.46 (9)	C13—C14—H14	119.9
O1^i^—Cd1—O3	84.22 (10)	C14—C15—C16	119.2 (3)
O3^ii^—Cd1—O3	72.80 (10)	C14—C15—C20	120.1 (3)
O4^ii^—Cd1—O3	107.31 (9)	C16—C15—C20	120.6 (3)
O2^i^—Cd1—O3	103.55 (9)	C17—C16—C15	121.3 (3)
O5—Cd1—O3	170.12 (9)	C17—C16—H16	119.4
N1—Cd1—C20^i^	119.82 (10)	C15—C16—H16	119.4
O1^i^—Cd1—C20^i^	27.56 (10)	C16—C17—C18	118.7 (3)
O3^ii^—Cd1—C20^i^	149.23 (10)	C16—C17—C21	120.9 (3)
O4^ii^—Cd1—C20^i^	104.05 (9)	C18—C17—C21	120.5 (3)
O2^i^—Cd1—C20^i^	27.15 (10)	C13—C18—C17	120.2 (3)
O5—Cd1—C20^i^	85.50 (10)	C13—C18—H18	119.9
O3—Cd1—C20^i^	96.99 (9)	C17—C18—H18	119.9
N1—Cd1—C19^ii^	112.60 (10)	O4—C19—O3	121.7 (3)
O1^i^—Cd1—C19^ii^	103.13 (9)	O4—C19—C13	120.5 (3)
O3^ii^—Cd1—C19^ii^	27.54 (9)	O3—C19—C13	117.8 (3)
O4^ii^—Cd1—C19^ii^	27.23 (9)	O4—C19—Cd1^ii^	61.48 (16)
O2^i^—Cd1—C19^ii^	151.79 (10)	O3—C19—Cd1^ii^	60.39 (17)
O5—Cd1—C19^ii^	96.88 (9)	C13—C19—Cd1^ii^	173.5 (2)
O3—Cd1—C19^ii^	89.19 (8)	O2—C20—O1	122.8 (3)
C20^i^—Cd1—C19^ii^	127.55 (10)	O2—C20—C15	118.6 (3)
C6—C1—C2	120.8 (3)	O1—C20—C15	118.6 (3)
C6—C1—H1	119.6	O2—C20—Cd1^i^	65.48 (18)
C2—C1—H1	119.6	O1—C20—Cd1^i^	58.25 (17)
C3—C2—C1	120.9 (3)	C15—C20—Cd1^i^	168.7 (2)
C3—C2—H2	119.5	C17—C21—H21A	109.5
C1—C2—H2	119.5	C17—C21—H21B	109.5
C4—C3—C2	118.4 (3)	H21A—C21—H21B	109.5
C4—C3—C7	121.3 (3)	C17—C21—H21C	109.5
C2—C3—C7	120.3 (3)	H21A—C21—H21C	109.5
C3—C4—C5	120.7 (3)	H21B—C21—H21C	109.5
C3—C4—H4	119.6	C9—N1—C8	105.8 (3)
C5—C4—H4	119.6	C9—N1—Cd1	127.0 (2)
C4—C5—C6	121.0 (3)	C8—N1—Cd1	126.6 (2)
C4—C5—H5	119.5	C9—N2—C7	107.9 (3)
C6—C5—H5	119.5	C9—N2—H2A	126.1
C1—C6—C5	118.1 (3)	C7—N2—H2A	126.1
C1—C6—C10	120.3 (3)	C12—N3—C11	107.5 (3)
C5—C6—C10	121.6 (3)	C12—N3—H3	126.3
C8—C7—N2	105.6 (3)	C11—N3—H3	126.3
C8—C7—C3	131.0 (3)	C12—N4—C10	104.8 (3)
N2—C7—C3	123.3 (3)	C20—O1—Cd1^i^	94.2 (2)
C7—C8—N1	109.3 (3)	C20—O2—Cd1^i^	87.4 (2)
C7—C8—H8	125.4	C19—O3—Cd1^ii^	92.07 (19)
N1—C8—H8	125.4	C19—O3—Cd1	121.7 (2)
N1—C9—N2	111.4 (3)	Cd1^ii^—O3—Cd1	107.20 (10)
N1—C9—H9	124.3	C19—O4—Cd1^ii^	91.29 (19)
			
C6—C1—C2—C3	−1.6 (5)	O3^ii^—Cd1—N1—C9	178.6 (3)
C1—C2—C3—C4	−0.4 (5)	O4^ii^—Cd1—N1—C9	142.9 (2)
C1—C2—C3—C7	178.2 (3)	O2^i^—Cd1—N1—C9	−5.3 (3)
C2—C3—C4—C5	1.6 (5)	O5—Cd1—N1—C9	68.9 (3)
C7—C3—C4—C5	−176.9 (3)	O3—Cd1—N1—C9	−108.5 (3)
C3—C4—C5—C6	−0.9 (6)	C20^i^—Cd1—N1—C9	−13.6 (3)
C2—C1—C6—C5	2.3 (5)	C19^ii^—Cd1—N1—C9	164.7 (3)
C2—C1—C6—C10	−176.8 (3)	O1^i^—Cd1—N1—C8	134.4 (3)
C4—C5—C6—C1	−1.1 (5)	O3^ii^—Cd1—N1—C8	−11.6 (3)
C4—C5—C6—C10	178.0 (3)	O4^ii^—Cd1—N1—C8	−47.3 (3)
C4—C3—C7—C8	−24.4 (5)	O2^i^—Cd1—N1—C8	164.5 (3)
C2—C3—C7—C8	157.1 (3)	O5—Cd1—N1—C8	−121.4 (3)
C4—C3—C7—N2	155.1 (3)	O3—Cd1—N1—C8	61.3 (3)
C2—C3—C7—N2	−23.4 (5)	C20^i^—Cd1—N1—C8	156.2 (3)
N2—C7—C8—N1	−0.8 (4)	C19^ii^—Cd1—N1—C8	−25.5 (3)
C3—C7—C8—N1	178.8 (3)	N1—C9—N2—C7	0.3 (4)
C1—C6—C10—C11	−167.0 (4)	C8—C7—N2—C9	0.3 (3)
C5—C6—C10—C11	13.8 (6)	C3—C7—N2—C9	−179.3 (3)
C1—C6—C10—N4	12.0 (5)	N4—C12—N3—C11	0.2 (5)
C5—C6—C10—N4	−167.2 (3)	C10—C11—N3—C12	−0.1 (4)
N4—C10—C11—N3	0.0 (4)	N3—C12—N4—C10	−0.2 (4)
C6—C10—C11—N3	179.1 (3)	C11—C10—N4—C12	0.1 (4)
C18—C13—C14—C15	0.0 (4)	C6—C10—N4—C12	−179.0 (3)
C19—C13—C14—C15	−177.5 (3)	O2—C20—O1—Cd1^i^	11.8 (4)
C13—C14—C15—C16	2.5 (4)	C15—C20—O1—Cd1^i^	−167.5 (2)
C13—C14—C15—C20	−174.4 (3)	O1—C20—O2—Cd1^i^	−11.0 (3)
C14—C15—C16—C17	−3.7 (5)	C15—C20—O2—Cd1^i^	168.3 (2)
C20—C15—C16—C17	173.2 (3)	O4—C19—O3—Cd1^ii^	−5.0 (3)
C15—C16—C17—C18	2.3 (5)	C13—C19—O3—Cd1^ii^	172.9 (2)
C15—C16—C17—C21	−177.8 (3)	O4—C19—O3—Cd1	−116.8 (3)
C14—C13—C18—C17	−1.4 (4)	C13—C19—O3—Cd1	61.2 (3)
C19—C13—C18—C17	176.1 (3)	Cd1^ii^—C19—O3—Cd1	−111.7 (2)
C16—C17—C18—C13	0.2 (4)	N1—Cd1—O3—C19	13.3 (2)
C21—C17—C18—C13	−179.7 (3)	O1^i^—Cd1—O3—C19	−130.6 (2)
C14—C13—C19—O4	−160.2 (3)	O3^ii^—Cd1—O3—C19	103.6 (3)
C18—C13—C19—O4	22.3 (4)	O4^ii^—Cd1—O3—C19	146.8 (2)
C14—C13—C19—O3	21.8 (4)	O2^i^—Cd1—O3—C19	−79.3 (3)
C18—C13—C19—O3	−155.7 (3)	C20^i^—Cd1—O3—C19	−106.1 (2)
C14—C15—C20—O2	20.9 (4)	C19^ii^—Cd1—O3—C19	126.1 (2)
C16—C15—C20—O2	−156.0 (3)	N1—Cd1—O3—Cd1^ii^	−90.31 (11)
C14—C15—C20—O1	−159.8 (3)	O1^i^—Cd1—O3—Cd1^ii^	125.78 (11)
C16—C15—C20—O1	23.3 (4)	O3^ii^—Cd1—O3—Cd1^ii^	0.0
C14—C15—C20—Cd1^i^	129.5 (11)	O4^ii^—Cd1—O3—Cd1^ii^	43.14 (12)
C16—C15—C20—Cd1^i^	−47.4 (13)	O2^i^—Cd1—O3—Cd1^ii^	177.06 (8)
N2—C9—N1—C8	−0.8 (4)	C20^i^—Cd1—O3—Cd1^ii^	150.26 (11)
N2—C9—N1—Cd1	170.7 (2)	C19^ii^—Cd1—O3—Cd1^ii^	22.49 (11)
C7—C8—N1—C9	1.0 (4)	O3—C19—O4—Cd1^ii^	5.0 (3)
C7—C8—N1—Cd1	−170.6 (2)	C13—C19—O4—Cd1^ii^	−172.9 (2)
O1^i^—Cd1—N1—C9	−35.4 (4)		

Symmetry codes: (i) −*x*+1, −*y*−1, −*z*+2; (ii) −*x*+1, −*y*, −*z*+2.

### Hydrogen-bond geometry (Å, °)

**Table:**

*D*—H···*A*	*D*—H	H···*A*	*D*···*A*	*D*—H···*A*
N2—H2A···N4^iii^	0.86	2.17	2.975 (4)	157.
N3—H3···O4^iv^	0.86	2.03	2.815 (4)	151.

Symmetry codes: (iii) −*x*+2, −*y*, −*z*+1; (iv) −*x*+1, −*y*+1, −*z*+1.
